# Downregulation of *Cyp7a1* by Cholic Acid and Chenodeoxycholic Acid in *Cyp27a1/ApoE* Double Knockout Mice: Differential Cardiovascular Outcome

**DOI:** 10.3389/fendo.2020.586980

**Published:** 2020-10-28

**Authors:** Line Zurkinden, Dmitri Sviridov, Bruno Vogt, Genevieve Escher

**Affiliations:** ^1^Department of Nephrology and Hypertension, Insel Gruppe, University Hospital Bern, Bern, Switzerland; ^2^Department of Biomedical Research, University of Bern, Bern, Switzerland; ^3^Institute of Biochemistry and Molecular Medicine, University of Bern, Bern, Switzerland; ^4^Baker Heart and Diabetes Institute, Melbourne, VIC, Australia; ^5^Department of Biochemistry and Molecular Biology, Monash University, Clayton, VIC, Australia

**Keywords:** bile acids, Western diet, sterol 27-hydroxylase, cholesterol absorption, LDL-cholesterol

## Abstract

Sterol 27-hydroxylase (CYP27A1) is a key enzyme in bile acids (BAs) biosynthesis and a regulator of cholesterol metabolism. *Cyp27a1/Apolipoprotein E* double knockout (DKO) mice fed with western diet (WD) are protected from atherosclerosis *via* up-regulation of hepatic *Cyp7a1* and *Cyp3a11*. Since feeding BAs ameliorates metabolic changes in *Cyp27a1* KO mice, we tested BAs feeding on the development of atherosclerosis in DKO mice. DKO mice were fed for 8 weeks with WD containing 0.1% cholic acid (CA) (WD-CA) or chenodeoxycholic acid (CDCA) (WD-CDCA). Atherosclerotic lesions, plasma lipoprotein composition and functionality, hepatic lipid content, BAs amount and composition, expression of genes involved in lipid metabolism and BA signaling in liver and intestine as well as intestinal cholesterol absorption were assessed. Hepatic *Cyp7a1* and *Cyp3a11* expression were reduced by 60% after feeding with both WD-CA and WD-CDCA. After feeding with WD-CA we observed a 40-fold increase in the abundance of atherosclerotic lesions in the aortic valve, doubling of the levels of plasma total and low density lipoprotein cholesterol and halving of the level of high density lipoprotein cholesterol. Furthermore, in these mice plasma cholesterol efflux capacity decreased by 30%, hepatic BA content increased 10-fold, intestinal cholesterol absorption increased 6-fold. No such changes were observed in mice fed with WD-CDCA. Despite similar reduction on *Cyp7a1* and *Cyp3a11* hepatic expression, CA and CDCA have a drastically different impact on development of atherosclerosis, plasma and hepatic lipids, BAs composition and intestinal absorption. Reduced cholesterol absorption contributes largely to athero-protection in DKO mice.

## Introduction

Sterol 27-hydroxylase (CYP27A1) is a mitochondrial enzyme ubiquitously expressed which belongs to the cytochrome P450 family. It catalyzes the hydroxylation of cholesterol at C27 to form 27-hydroxycholesterol (27-OHC) and cholestenoic acid (1, 2). CYP27A1 plays a major role in cholesterol homeostasis. In the liver, it is involved in pathways of cholesterol metabolism due to its role in bile acids (BAs) biosynthesis. CYP27A1 catalyzes the first step of the alternative pathway of BAs synthesis, as well as intermediate steps of the classical pathway initiated by cholesterol 7α-hydroxylase (CYP7A1) (3, 4). CYP7A1 acts not only as the key enzyme for cholesterol metabolism, it is also the rate-limiting enzyme for BAs biosynthesis in the liver (5). In extrahepatic tissues, CYP27A1 is involved in reverse cholesterol transport, since overexpression in macrophages enhances cholesterol efflux, the transfer of cholesterol to an acceptor in plasma, both *in vitro* and *in vivo* (6–8). Finally, CYP27A1 regulates cholesterol biosynthesis, as 27-OHC was shown to act *in vitro* as negative feedback regulator on the rate limiting enzyme of cholesterol biosynthesis, the 3-Hydroxy-3-Methylglutaryl-Coenzym-A-Reductase (HMGCR) (9).

Given the role of CYP27A1 in cholesterol efflux and BAs synthesis, we investigated the effect of *Cyp27a1* deficiency on the development of atherosclerosis in *Apolipoprotein E* (*ApoE*) knock out (KO) mice, anticipating that atherosclerotic lesions would be increased in *Cyp27A1* KO/*ApoE* KO double knockout (DKO) mice. *ApoE* KO mice develop accelerated atherosclerosis, especially when fed with pro-atherogenic western diet (WD), but the atherosclerotic phenotype of *ApoE* KO was unexpectedly abolished in DKO mice fed with WD (10). This surprising phenotype was attributed to reduced plasma total cholesterol (TC) and low density lipoprotein-cholesterol (LDL-C), elevated high density lipoprotein-cholesterol (HDL-C), compensatory increase in cholesterol catabolism *via* sterol 7α-hydroxylase (*Cyp7a1*) and sterol 12α-hydroxylase (*Cyp8b1*), increased expression of *Cyp3a11* minimizing the production of toxic bile alcohols and cholesterol, and increased cholesterol excretion (10).

BAs are physical detergents greatly facilitating the intestinal absorption of lipids and vitamins (11). These amphiphilic molecules can increase cholesterol solubility to promote the intestinal absorption of cholesterol (12). Furthermore, BAs are the major metabolites of cholesterol excreted through the bile out of the liver (13). They are also critical signaling molecules mediating nutrient homeostasis and energy expenditure (14). BAs are known to regulate their own synthesis and transport *via* farnesoid X receptor (FXR) in the liver and intestine. Different BAs have differential effects on bile acid signaling. Chenodeoxycholic acid (CDCA) is a much more potent FXR agonist than cholic acid (CA) (15). In studies in mice however, CA is a more potent FXR activator then CDCA since CDCA is rapidly converted into muricholic acids (MCAs) (16). MCAs have different signaling and lipid solubilizing properties compared to CDCA, they act as FXR antagonists and do not activate G protein-coupled bile acid receptor 1 (TGR5) (17). CA is an important mediator of the negative feedback regulation of BAs biosynthesis in mice (18). Loss of CA from the bile acid pool causes a decrease in the hepatic expression of a negative regulatory factor (SHP), which in turn causes a loss of repression of the *Cyp7a1* gene and an increase in biosynthetic output (19). In the intestine, fibroblast growth factor 15 (FGF15) expression is induced by bile acids acting on the FXR (20). In *Cyp27a1* KO mice, CA feeding was shown to reverse the hepatomegaly, to suppress the up-regulation of hepatic *Cyp7a1* and *Cyp8b1* and to restore intestinal cholesterol absorption to normal levels after 10 days of supplementation with 0.1% CA (21). The hepatomegaly was also almost fully reversed when CDCA was fed (21).

We hypothesized that, like in *Cyp27a1* KO mice, feeding DKO mice with 0.1% CA or 0.1% CDCA will restore the hepatomegaly, suppress the up-regulation of the hepatic cytochromes and increase intestinal cholesterol absorption in DKO mice. These metabolic changes should lead to an increase in atherosclerosis development. To prove this, we fed DKO mice with a WD containing either 0.1% CA (WD-CA) or 0.1% CDCA (WD-CDCA) and studied the effect of exogenous BAs supplementation on plasma composition, efflux cholesterol capacity (CEC), expression of hepatic cytochromes, atherosclerotic plaque formation and intestinal cholesterol absorption.

## Methods

### Chemicals

Cholic acid (CA) (C1129), chenodeoxycholic acid (CDCA) (C9377), Oil red O (O0625), hematoxylin solution Gill no. 3 (GHS316), and eosin Y solution aqueous (HT110216) were from Sigma-Aldrich (St Louis, MO, USA); oligonucleotides were from Microsynth (Balgach, Switzerland); the hexanucleotide mix was from Roche Diagnostics (Mannheim, Germany). [4-^14^C] Cholesterol and [22,23-^3^H] β-sitosterol were obtained from American Radiolabeled Chemical, Inc. (St Louis, MO, USA).

### Animals

Animal experimentation was approved by the Ethics Committee for Animal Experiments of the Veterinary Administration of the Canton of Berne, Switzerland (BE64/11 and BE 48/14), and conformed to the rules of the Swiss Federal Act on Animal Protection. Experiments were carried out at the central animal facility of the University of Bern.

The Bern *Cyp27a1^+/^*^−^ mouse-breeding colony (provided by S.K.E., University of California, San Francisco, CA, USA), was crossed with *ApoE*^−^*^/^*^−^ (*ApoE* KO) mice (Charles River Laboratories (Sulzfeld, Germany). The resulting *Cyp27a1*^−^*^/^*^−^/*ApoE*^−^*^/^*^−^ double knock out (DKO) mouse and *Cyp27a1^+/+^*/*ApoE* (*ApoE* KO) on C57BL/6J background were bred and genotyped as described (22).

Mice were maintained under 12-h dark–light cycles with unrestricted access to food and water. At the age of 5 weeks, males (n=18) were fed a WD containing 21% fat and 0.15% cholesterol (D-12079B; Provimi Kliba AG). A week later, animals were divided into 3 groups and fed for 8 weeks either WD, or WD containing 0.1% CA (WD-CA) or 0.1% CDCA (WD-CDCA). Animals were then placed in metabolic cages for 4 days. Urine and feces were collected over 24 h for BAs quantification on the 4^th^ day, after 3 days of pre-adaptation. Mice returned to their cage for 2 days and were then starved for 4 h and sacrificed by pentobarbital injection (300 mg/kg, pentobarbital sodium, USP; Abbott Laboratories, North Chicago, IL, USA). The animals were weighed and blood was collected into a tube containing 20 to 50 U heparin, centrifuged at 4°C for 15 min at 13,000 rpm and stored at −20°C until analyzed. Organs were removed, weighed and washed with PBS. One part was fixed in formalin at 4°C, the other frozen in liquid nitrogen and stored at −70°C until processed. The heart and aorta were fixed in 4% paraformaldehyde for 24 h, transferred to PBS, and stored at 4°C until used.

A second cohort of mice was used for cholesterol absorption. Eight weeks old mice (n = 6–7) were fed for 2 weeks with WD, then for another 2 weeks with WD-CA or WD-CDCA. At the end of each food regimen, cholesterol absorption was assessed by dual isotope incorporation. Gavage was performed with 100 μl soybean oil containing 0.1 μCi [4-^14^C] cholesterol and 0.2 μCi [22,23-^3^H] β-sitosterol. After gavage, each mouse was individually housed in a cage covered with Whatman paper and had free access to food and water. Feces were collected daily for 3 days. Samples were extracted by saponification with 5M KOH in 50% ethanol and heated at 80°C for 1 h. Lipids were extracted by Folch method. The extracted lipids were transferred into scintillation vials and isotopes were counted in β-counter. The percentage of cholesterol absorption was calculated as follows:

$$\frac{\frac{14C}{3H}dose\;ratio-\frac{14C}{3H}faeces\;ratio}{\frac{14C}{3H}dose\;ratio}\times \;100$$

### Plasma and Tissue Biochemistry

Plasma TC, triglycerides (TG), LDL-C+VLDL-C and HDL-C were quantified with a kit from Wako Chemicals GmbH (Neuss, Germany), and cholesterol and cholesteryl esters (CE) with a CE quantification kit from Calbiochem-Merck (Millipore, Zug, Switzerland). Apolipoprotein A-1 (ApoA-1) was measured by enzyme-linked immunosorbent assay kit from cloud-clone corp. (Houston, TX, USA). In liver and feces homogenates, TG were assayed using a TG quantification kit (BioVision, Mountain View, CA, USA), and cholesterol and cholesterol esters with quantification kit (Calbiochem-Merck Millipore, Zug, Switzerland).

BAs were quantified by gas chromatography-mass spectrometry (GC-MS) in liver (100 mg), bile (1 µl), urine (200 µl) and feces (10–250 mg) with 400 ng 23-Nordeoxychcolic as recovery standard, as previously done (10, 23). BAs from bile and urine were diluted in 5 ml saline and extracted with Sep-Pack (Waters Corp., Milford, MA, USA). Hepatic BAs were extracted successively with 2 ml ethanol 100%, 2 ml ethanol 80%, and 2 ml methanol at 100°C as described by Locket and Gallaher (24). The supernatant from each extraction was collected and pooled, evaporated, re-suspended in 5 ml water and extracted on a Sep-Pak C18 cartridge. BAs from feces were extracted with 10 ml butanol/water (1:1, vol/vol) overnight and the supernatant was collected following centrifugation for 10 min at 3000 rpm. All the samples were submitted to subsequent solvolysis, hydrolysis and derivatization (23).

### Histology

For *en face* analysis, the aorta was opened longitudinally, stained with 5% Sudan IV (Carl Roth, Karlsruhe, Germany), and pinned onto a white Styrofoam surface for imaging.

Lesions of the aortic valve were analyzed in paraffin embedded sections of 10 µm at 20 µm intervals for 700 µm and stained with hematoxylin and eosin (H&E). Lesion areas were quantified in a blinded manner by image analysis software (ImageJ; NIH, Bethesda, MD, USA) as described (10). Sections of paraffin embedded liver (10 µm) were stained with H&E.

Jejunums were embedded in OCT. Frozen sections of 5 μm were air-dried, stained with Oil red O for 10 min and rinsed with tap water for 30 min. For analyzing, 10 frames per slide were captured on NIS-Elements F2.20 microscope (Nikon Ltd, UK) at 40× magnification. Quantification of the specific staining was performed in a blinded manner, using the positive pixel algorithm of ImageJ software.

### RNA Extraction and Real-Time PCR in Liver and Intestine

RNA was extracted with TRIzol Reagent (Thermo Fisher scientific) from 100 mg liver and 0.5 cm intestine. Reverse transcription was performed with 2 µg RNA in a reaction containing 100 U SuperScript Reverse Transcriptase type II (Roche, Basel, Switzerland). Real-time PCR was performed with 100 ng cDNA/reaction. The following primers were used: CYP3A11 (Mm00731567_m1), HMGR (*hmgcr*) (Mm01282499_m1), PXR (Nr1i) (Mm01344139_m1), fatty acid synthase (Fasn) (Mm00662319_m1), SREBP2 (Srebf2) (Mm01306292_m1), diacylglycerol acyltransferase (Dgat1) (Mm00515643_m1), CYP7A1 (Mm0048T4152_m1), CYP27A1 (Mm00470430_m1), CYP8B1(Mm00501637_s1), LXRα (Mm00443451_m1), FXR (Nr1h4) (Mm00436419_m1), SR-B1 (Mm00450234_m1), LDLR (Mm01177349_m1), ABCA1 (Mm00442646_m1), ABCG5 (Mm00446241_m1), ABCG8 (Mm00445970_m1), SREBP-1c (Srebf-1) (Mm00550338_m1), SHP (Nr0b2) (Mm00442278_m1), and BSEP (Abcb11) (Mm00445168_m1), FGFR4 (Mm01341852_m1), FGF15 (Mm00433278_m1). The other primers were obtained from Microsynth (Switzerland) and probes from Roche Diagnostics (Germany) and are listed in **Supplementary Table 1**. β-Actin (AM1720) was used as internal standard. Quantification was performed by the relative quantification method, using DKO mice fed with WD as calibrator.

### Cholesterol Efflux

Cholesterol efflux was performed as described previously (25), using the plasma obtained from the different groups of mice as acceptor. Briefly, RAW264.7 cells were plated in 12-wells plates and grown in RPMI supplemented with 100 µg/ml penicillin-streptomycin, 1% L-glutamine and 10% fetal bovine serum (FBS) at 5% CO_2_ and 100% humidity. Cells were labeled for 48 h with [^3^H]-cholesterol (Anawa, Switzerland), washed 3 times with PBS and equilibrated O/N in OptiMem. Cells were washed 2 times and further incubated for 2 h in the presence or absence of 2% plasma obtained from experimental animals. The medium was collected, centrifuged for 15 min at 4°C at 2,500 rpm. Cells were harvested, resuspended in 1 ml of distilled water and radioactivity was counted in 100 µl aliquots. Cholesterol efflux was calculated as percentage of labeled cholesterol released to the medium divided by the amount of total cholesterol in the medium and cells in each well.

### Statistical Analysis

To determine statistically significant differences, t-test or one-way ANOVA was used, followed by Tukey or Dunn’s *post hoc* test for multiple comparisons. Correlations between the parameters were tested with a Pearson’s Rank correlation tests. The null Hypothesis was rejected if P<0.05.

## Results

### Effect of WD-CA and WD-CDCA on Body and Organ Weights

The effect of 8 weeks’ supplementation of WD with CA or CDCA on organs weight on DKO mice is shown in **Table 1**. When compared to WD, WD-CA had no effect on body weight (BW) and liver weight, but slightly increased lung weight. In contrast, WD-CDCA led to a significant reduction of liver weight, without affecting BW and lung weight. The weights of spleen, kidney and brain were unaffected by exogenous BA feeding (data not shown).

**Table 1:** Effect of CA and CDCA on body and organ weight, and lipid composition in plasma, liver and bile.

Parameter		WD	WD-CA			WD-CDCA	P
		n=6	n= 6			n= 6		
**Weight**								
BW (g)	27.5 ± 1.2			26.9 ± 1.8		26.9 ± 1.8	NS	
Liver (g)	1.8 ± 0.4			1.8 ± 0.2		1.4 ± 0.2*	0.009	
Liver/BW	6.5 ± 1.5			6.7 ± 1		5.4 ± 0.7	0.0334	
Lung (g)	0.16 ± 0.02			0.18 ± 0.02*		0.16 ± 0.01	0.0112	
Lung/BW	5.6 ± 0.5			6.8 ± 1*		5.7 ± 0.4	0.0269	
**Plasma**								
TC (mmol/l)	8.8 ± 1.3			21.8 ± 4.4**		9.4 ± 1.9	0.0004	
HDL-C (mmol/l)	0.81 ± 0.07			0.45 ± 0.04**		0.65 ± 0.15	0.0006	
LDL+VLDL-C (mmol/l)	7.8 ± 1.9			15.2 ± 4.0***		8.5 ± 1.9	0.0005	
TG (mmol/l)	1.9 ± 0.7			2.1 ± 0.9		1.3 ± 0.8	NS	
ApoA-I (mg/dl)	93.4 ± 40.8			131.7 ± 39.2		113.9 ± 50	NS	
AST (IU/l)	18 ± 6			32 ± 8**		n.m.	0.0049	
ALT (IU/l)	227 ± 44			418 ± 79**		n.m.	0.0049	
Cholesterol efflux (%)	3.1 ± 0.3			2.4 ± 0.5*		3.1 ± 0.7	0.0458	
**Liver**								
TC (μg/mg)	6.7 ± 1.3			16.2 ± 3.6***		7.4 ± 2.7	0.0001	
CE (μg/mg)	1.3 ± 0.8			2.9 ± 0.8**		1.5 ± 0.5	0.0011	
TG (nmol/mg)	61.6 ± 13.8			28.2 ± 9.9***		37.2 ± 10.8**	0.0005	
**Bile**								
TC (mmol/10μl)	1.6 ± 0.7			11.1 ± 3.1***		3.9 ± 1.1	0.0001	

Results are presented as means ± SEM.

For statistical significance, *P < 0.05, **P < 0.01, ***P < 0.001 vs. WD.

NS, not significant.

n.a., not applicable.

### Impact of WD-CA and WD-CDCA on Atherosclerosis Formation

**Figure 1:**
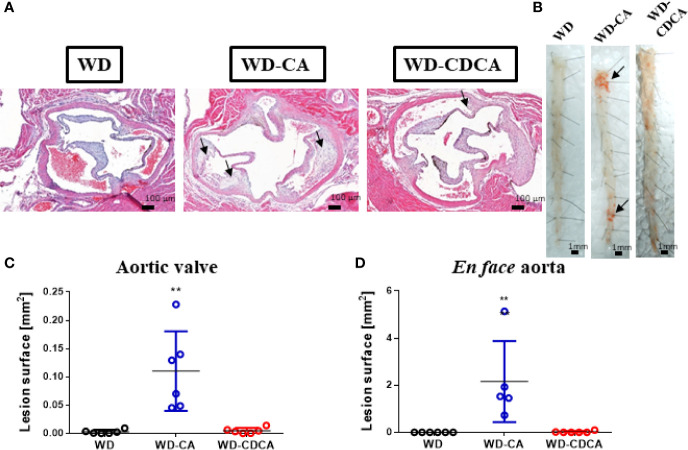
Effect of WD, WD-CA, and WD-CDCA on the development of atherosclerosis in DKO mice. Mice were fed for two months with the appropriate diet and atherosclerosis was quantified in aortic valve sections and en face. **(A)** Representative photomicrographs of aortic valve in paraffin-embedded sections stained with H&E. Scale bars 200 µm. **(B)** Representative en face Sudan IV staining of aorta. Scale bars 1 mm. **(C)** Quantification of atherosclerotic lesions in aortic valve [mm2]. **(D)** Quantification of atherosclerotic lesions in en face aorta [mm2]. Arrows indicate atherosclerotic plaques. Data are means ± SEM. For statistical significance, **P < 0.01.

Atherosclerotic plaque development was quantified in aortic valve sections (**Figures 1A, C**) and *en face* (**Figures 1B, D**) in animals fed for 8 weeks with WD, WD-CA or WD-CDCA. As previously reported DKO mice failed to develop atherosclerosis (10). After feeding with WD-CA, atherosclerotic plaque increased in aortic valve sections from 0.0027 ± 0.0014 to 0.110 ± 0.028 mm^2^ (mean ± SEM) (P<0.001) (**Figure 1C**). In *en face* aorta, lesions increased from 0.00 ± 0.00 to 2.16 ± 0.77 mm^2^ (mean ± SEM) (P<0.0001) (**Figure 1D**). In contrast to WD-CA, WD-CDCA had almost no effect on atherosclerosis development in aortic valve (**Figure 1C**) and *en face* aorta when compared to WD (**Figure 1D**).

### Influence of WD-CA and WD-CDCA Diets on Lipid Composition of Plasma, Liver, and Bile

Plasma lipoprotein profile is shown in **Table 1**. After WD-CA diet, there was a ~2-fold increase of TC (P<0.01) and LDL-C+VLDL-C (P<0.001), while HDL-C reduced almost 2-fold (P<0.01). TG and apoA-1 levels were unchanged. WD-CDCA had no effect on plasma lipoprotein composition when compared to WD (**Table 1**). ALT and AST increased in mice fed with WD-CA compared with WD only (P<0.001) but could not be measured in the WD-CDCA group because of interference of this BA with the assay.

CA and CDCA feeding also modified hepatic lipids composition. TC and CE content doubled after WD-CA feeding (P<0.0001 and P<0.01) but remained constant with WD-CDCA feeding when compared to WD alone (**Table 1**). Both WD-CA and WD-CDCA feedings reduced liver TG content by about 50% (P<0.01 and P<0.001) (**Table 1**). In the bile, the amount of TC increased 7 fold when mice were fed with WD-CA and 2.5 fold in mice fed with WD-CDCA (P<0.001) (**Table 1**).

### Effect of WD-CA and WD-CDCA Diets on Plasma Efflux Capacity

To test the effect of BAs on HDL functionality, cholesterol efflux was performed in RAW 264.7 cells, using plasma obtained from experimental mice. Cholesterol efflux was reduced when macrophages were incubated with plasma from mice fed with WD-CA (P<0.05) (**Table 1**). Cholesterol efflux correlated with plasma HDL-C (R^2 =^ 0.373, r=0.611, P<0.001).

### Contribution of WD-CA and WD-CDCA Feeding to Changes in BAs Amount and Composition

Similar amounts of CA and CDCA were ingested, as calculated from food intake when mice were in metabolic cages (**Table 2**).

**Table 2:** Quantification of BA intake and BA composition in liver, bile juice, faeces and urine.

BA		WD	WD-CA	WD-CDCA	One way Anova
		n= 6	n= 6	n= 6	P
**Food**	CA	none	9.38 ± 2.19	none	n.a.
**[μmol/24h]**	CDCA	none	none	10.64 ± 1.5	n.a.
**Liver**					
**[nmol/g]**	BAs total	7.45 ± 1.26	91.95 ± 25.89**	48.9 ± 8.90*	0.0002
	CA	2.26 ± 0.55	71.17 ± 22.44*	1.15 ± 0.26	0.0001
	CDCA	0.27 ± 0.03	0.18 ± 0.01	7.92 ± 2.70*	0.0001
	αMCA	0.37 ± 0.04	0.48 ± 0.03	10.74 ± 2.13**	0.0001
	βMCA	2.60 ± 0.74	1.50 ± 0.41	11.61 ± 1.40***	0.0001
	ωMCA	0.80 ± 0.07	0.72 ± 0.12	3.77 ± 0.69	0.0003
	UDCA	0.42 ± 0.06	0.08 ± 0.04	12.63 ± 3.78**	0.0041
	DCA	0.57 ± 0.08	4.97 ± 0.98**	0.72 ± 0.05	0.0002
	LCA	ND	ND	ND	ND
	HDCA	0.20 ± 0.02	0.17 ± 0.05	0.35 ± 0.07	NS
**Bile juice**					
**[pmol/μl]**	Bas total	81.30 ± 4.05	172.00 ± 46.23	176.50 ± 9.61*	0.029
	CA	10.73 ± 2.14	90.68 ± 35.43	14.81 ± 0.84	NS
	CDCA	8.39 ± 0.31	7.67 ± 0.12	21.26 ± 4.472*	0.0138
	αMCA	7.73 ± 0.20	22.98 ± 10.03	28.57 ± 1.64	NS
	βMCA	10.53 ± 0.47	9.64 ± 0.52	50.18 ± 4.80***	0.0001
	ωMCA	13.42 ± 2.17	10.44 ± 0.79	20.48 ± 2.57	0.0221
	UDCA	9.46 ± 0.19	8.36 ± 0.14	18.13 ± 1.91	0.0001
	DCA	7.14 ± 0.17	7.57 ± 0.35	6.68 ± 0.12	NS
	LCA	8.50 ± 0.18	7.44 ± 0.13	7.81 ± 0.15	NS
	HDCA	8.38 ± 0.16	7.27 ± 0.14	8.57 ± 0.20	NS
**Feces**					
**[nmol/24h]**	Bas total	253.0 ± 18.7	357 ± 49	3044 ± 269.3**	0.0001
	CA	40.1 ± 5.3	158.9 ± 34.2**	59.9 ± 9.4	0.0008
	CDCA	3.2 ± 0.3	0.4 ± 0.1	68.6 ± 12.8***	0.0001
	αMCA	10.1 ± 2.0	1.7 ± 0.3	532.1 ± 101.6***	0.0001
	βMCA	17.8 ± 5.0	8.6 ± 2.5	785.2 ± 96.8*	0.0001
	ωMCA	122.2 ± 14.5	40.2 ± 8.2	1520.0 ± 196.2***	0.0001
	UDCA	4.5 ± 0.7	2.9 ± 0.4	58.2 ± 8.0**	0.0009
	DCA	14.3 ± 1.7	140.8 ± 15.7***	23.0 ± 2.1	0.0001
	LCA	22.7 ± 6.0	0.4 ± 0.1**	10.2 ± 2.9	0.0037
	HDCA	18.2 ± 2.3	2.7 ± 0.3	86.3 ± 13.5***	0.0001
**Urine**					
**[nmol/24h]**	Bas total	3.22 ± 0.83	6.21 ± 2.32	9.90 ± 4.36	NS
	CA	1.41 ± 0.58	6.80 ± 3.74	0.12 ± 0.07	NS
	CDCA	0.03 ± 0.01	ND	0.23 ± 0.13	NS
	αMCA	0.02 ± 0.01	0.01 ± 0.01	0.27 ± 0.20	NS
	βMCA	0.07 ± 0.03	0.02 ± 0.01	0.34 ± 0.20	NS
	ωMCA	0.97 ± 0.36	0.69 ± 0.19	4.32 ± 2.77	NS
	UDCA	0.06 ± 0.02	0.05 ± 0.01	1.43 ± 0.84	NS
	DCA	0.08 ± 0.04	0.05 ± 0.02	0.01 ± 0.00	NS
	LCA	0.09 ± 0.02	0.10 ± 0.03	0.02 ± 0.01*	0.0089
	HDCA	0.15 ± 0.04	0.03 ± 0.02	0.07 ± 0.03	NS

Results are presented as means ± SEM.

For statistical significance, *P < 0.05, **P < 0.01, ***P < 0.001 vs. WD.

NS not significant.

n.a. not applicable.

ND not detectable.

BA content was measured in liver, bile, feces and urine (**Table 2**). Mice fed with WD-CA had markedly elevated BAs in liver (P<0.01) whereas those fed with WD-CDCA had increased total BAs in liver (P<0.05), bile (P<0.05) and feces (P<0.01) when compared to those fed with WD alone. The amount of total BAs did not change in urine with both diets (**Table 2**).

The analysis of bile acid composition revealed in animals fed with WD-CA a 32-fold increase of CA in the liver (P<0.05) and 4-fold increase in feces (p<0.01), representing 0.76 ± 0.23% and 1.69 ± 0.36% respectively of the ingested CA (**Table 2**). Content of the secondary BA, deoxycholic acid (DCA) metabolized from CA, also increased 10-fold in the liver (P<0.01) and feces (P<0.001), contributing 1to the excretion of 0.05 ± 0.01% and 1.50 ± 0.16% respectively of the ingested CA. The content of LCA markedly decreased in feces but represented only 0.005 ± 0.002% of the ingested CA (P<0.01) (**Table 2**). No significant changes were observed in urine in which BAs excretion was 50 fold lower when compared to feces P<0.0001).

In mice fed with WD-CDCA, CDCA content increased more than 20 fold in liver and feces (P<0.05 and P<0.001) and 2.5 fold in the bile (P<0.05), but urinary excretion remained constant (**Table 2**). The increase in total BA content in the liver was not only due to the accumulation of CDCA, but also to the presence of α-muricholic acid (αMCA), β-muricholic (βMCA), ω-muricholic (ωMCA), and UDCA, representing 0.07 ± 0.03%, 0.10 ± 0.02%, 0.11 ± 0.01%, 0.04 ± 0.01% and 0.12 ± 0.04% of the ingested CDCA (**Table 2**). In the liver, αMCA increased more 30 fold (P<0.001), βMCA and ωMCA increased 4 fold (P<0.001 and P<0.01) and combined the 3 MCAs constituted more than half of the BAs pool. The same tendency was seen in bile. The most pronounced changes in BA composition was observed in feces where more than 90% of BAs consisted of MCAs, whereas in mice fed with WD, they represented less than 60% of total BA excretion. Ursodeocycholic acid (UDCA) and hyodeoxycholic acid (HDCA) content were also significantly increased (P<0.01 and P < 0.001), but they represented a smaller proportion of BA (**Table 2**). In total, 28.02 ± 3.18% of the ingested CDCA was excreted (as CDCA but also as UDCA, LCA, HDCA and MCAs) in feces. The only change in urinary BA composition was a slight decreased (P<0.05) in LCA.

### Effect of WD-CA and WD-CDCA Diets on Liver Histology

**Figure 2:**
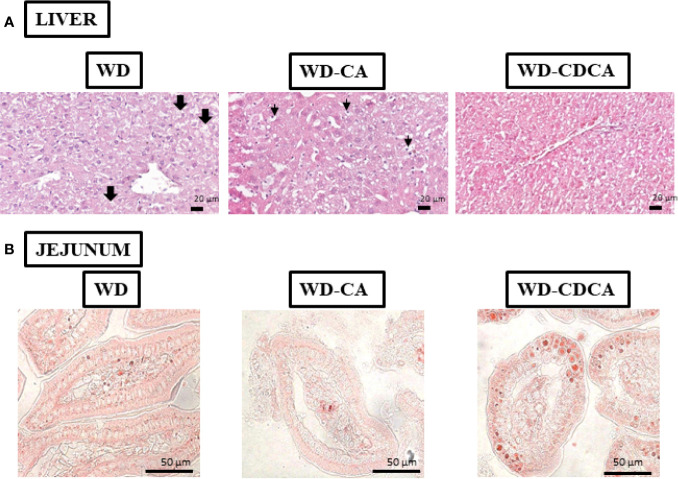
Effect of WD, WD-CA, and WD-CDCA on liver and jejunum. **(A)** Representative photomicrographs of H&E staining of liver **(A)** and Oil red O staining of jejunum **(B)** sections of DKO mice fed for 2 months with WD, WD-CA or WD-CDCA. Scale bars 20 µm for liver, 50 µm for ileum. In liver, thick arrows indicate ballooning degeneration of hepatocytes, and thin arrows designate cleared hepatocytes.

Histology analyses of the liver revealed that all mice fed with WD suffered from macrovesicular steatosis. The condition was slightly more severe in animals fed with WD-CA (**Figure 2A**). There was some microvesicular steatosis specific for mice fed with WD and WD-CA but not WD-CDCA. Ballooning degeneration of hepatocytes was observed in mice fed WD only. With WD-CA, a large quantity of cleared hepatocytes was present (**Figure 2A**).

### Impact of WD-CA and WD-CDCA Diets on Expression of Liver Genes Involved in Lipid Metabolism and Inflammation

Gene expression results are summarized in **Table 3**. First, we assessed the effect of CA and CDCA on the expression of genes involved in cholesterol metabolism. The mRNA for *Cyp7a1* and *Cyp3a11*, two genes involved in BAs metabolism and detoxification, decreased 3-fold (P<0.01) and 2-fold (P<0.001) respectively with both diets. Expression of *Cyp8b1*, a key determinant of CA production, decreased more than 10-fold in mice fed with WD-CA (P<0.001) and did not change significantly in those fed with WD-CDCA. The expression of genes involved in cholesterol homeostasis, HMGCR *(Hmgcr)*, sterol regulatory element-binding protein 2 (*Srebf2*) and low density lipoprotein receptor (*Ldlr*) significantly decreased in animals fed with WD-CA (P< 0.01, P<0.001 and P<0.05). The effect was specific for CA except for *Srebf2* expression of which was also reduced by WD-CDCA (P<0.01) (**Table 3**).

**Table 3:** Effect of CA and CDCA on expression of genes involved in lipid metabolism and inflammation in the liver.

Role	Gene	WD	WD-CA	WD-CDCA	One way Anova
		n=6	n=6	n= 6	P
**Cholesterol homeostasis**	*Cyp7a1*	1.04 ± 0.32	0.37 ± 0.18**	0.38 ± 0.28**	0.0006
	*Cyp3a11*	1.01 ± 0.19	0.48 ± 0.17***	0.48 ± 0.12***	0.0001
	*Cyp8b1*	1.01 ± 0.17	0.08 ± 0.04***	0.65 ± 0.18	0.0008
	*Hmgcr*	1.06 ± 0.40	0.13 ± 0.06**	0.62 ± 0.36	0.0051
	*Srebf2* (SREBP2)	1.01 ± 0.16	0.27 ± 0.04***	0.68 ± 0.19**	0.0001
	*Ldlr*	1.07 ± 0.39	0.38 ± 0.10*	0.94 ± 0.25	0.0153
**BAs signaling**	*Slco1a1* (OATP1)	1.01 ± 0.15	0.91 ± 0.34	1.19 ± 0.29	NS
	*Slc10a1* (NTCP)	1.10 ± 0.52	0.44 ± 0.25	2.07 ± 0.71*	0.0003
	*Slc51b* (OSTb)	1.09 ± 0.50	6.46 ± 2.61**	1.26 ± 0.69*	0.0047
	*Abcb11* (BSEP)	1.01 ± 0.12	0.85 ± 0.19	1.11 ± 0.21	NS
	*Fgf4*	1.00 ± 0.12	0.79 ± 0.13*	0.96 ± 1.14	0.0287
**Cholesterol transport**	*Abca1*	1.02 ± 0.20	1.50 ± 0.21***	1.43 ± 0.10**	0.0005
	*Nr1h3* (LXRα)	1.01 ± 0.13	0.73 ± 0.15**	0.93 ± 0.08	0.0034
	*Abcg1*	1.02 ± 0.23	3.06 ± 0.41***	1.52 ± 0.22*	0.0001
	*Abcg5*	1.03 ± 0.24	2.27 ± 0.71*	1.50 ± 0.19*	0.013
	*Abcg8*	1.03 ± 0.29	2.37 ± 0.66***	1.75 ± 0.40*	0.0007
	*Scarb1* (SR-B1)	1.02 ± 0.22	1.20 ± 0.22	1.09 ± 0.21	NS
	*Cav-1*	1.01 ± 0.15	0.50 ± 0.31**	0.58 ± 0.21*	0.0034
**Nuclear receptors**	*Nr1i2* (PXR)	1.01 ± 0.19	0.80 ± 0.26	1.09 ± 0.17	NS
	*Nr1h4* (FXR)	1.06 ± 0.35	0.79 ± 0.27	1.39 ± 0.31	0.0146
	*Nr0b2* (SHP)	1.31 ± 1.29	3.68 ± 1.07**	2.97 ± 0.89*	0.0059
	*Vdr*	1.09 ± 0.45	5.70 ± 3.06**	1.44 ± 0.50	0.0008
	*Rxrα*	1.02 ± 0.22	0.77 ± 0.23	1.19 ± 0.27	0.0302
**Fatty acids and TG biosynthesis**	*Pparγ*	1.04 ± 0.32	0.31 ± 0.06**	0.88 ± 0.17	0.0024
	*Fasn*	1.02 ± 0.24	0.25 ± 0.08***	0.88 ± 0.36	0.0085
	*Dgat1*	1.01 ± 0.14	0.67 ± 0.15**	1.03 ± 0.12	0.0005
	*Srebf1 (SREBP-1c)*	1.05 ± 0.35	1.54 ± 0.57	2.33 ± 0.39***	0.0007
**Inflammation**	*Tnfα*	1.06 ± 0.38	2.34 ± 0.80**	0.94 ± 0.34	0.0008
	*Il6*	1.13 ± 0.60	0.53 ± 0.30	0.73 ± 0.36	NS

Results are presented as means ± SD.

For statistical significance, *P < 0.05, **P < 0.01, ***P < 0.001 vs. WD.

NS not significant.

Among BAs transporters investigated, expression of *Slc51b*, the gene for organic solute transporter β (OSTβ), increased more than 5-fold with WD-CA (P<0.01), whereas expression of *Slc10a1*, the gene for sodium/bile acid cotransporter (NTCP), increased specifically with CD-CDCA (P<0.05). The expression of *Slco1a1* (gene for organic-anion-transporter protein [OATP1]), *Gpbar1* (gene for G protein-coupled bile acid receptor 1 [TGR5]) and *Abcb11* (gene for the bile salt export pump [BSEP]) remained unchanged (**Table 3**). The reasons for an inconsistency in the effects of BAs on the expression of OSTβ and *Abcb11* when both genes are activated by FXR are not clear, but are likely attributed to *Cyp27a1* deficiency.

For genes involved in cholesterol transport, we observed a 3-fold increase in *Abcg1* expression (P<0.001), a more than 2-fold increase in expression of *Abcg8* (P<0.001) and *Abcg5* (P<0.05), and a slight increase in expression of *Abca1* (P<0.001) in WD-CA fed animals (**Table 3**). The expression of *Nr1h3* (gene for Liver X receptor [LXR]) and *Cav-1* (gene for caveolin-1) were reduced (P<0.01), and the expression of *Scarb1* (SR-B1) remained unchanged. Similar, but less pronounced effects on the exception of *Nr1h3* were observed with WD-CDCA (**Table 3**).

WD-CA and WD-CDCA diets did not change the expression levels of the nuclear receptors *Nrli2* (gene for PXR) and *Nrlh4* (gene for FXR) but increased expression of *Nr0b2* (gene for small heterodimer protein [SHP]) (P<0.01). WD-CA diet enhanced expression of *Vdr* (P<0.01) and decreased expression of *Ffg4* (P<0.05) (**Table 3**).

Among genes involved in fatty acid and triglycerides synthesis (**Table 3**), expression of the transcription factor peroxisome proliferator activated receptor gamma (*Pparγ*) and fatty acid synthase (*Fas*) decreased 3- to 4-fold (P<0.01 and P<0.001), and expression of diglyceride acyltransferase (*Dgat-1*) decreased slightly (P<0.01) in mice fed with WD-CA but not with WD-CDCA. In contrast, the expression *of Srebf1*, the gene for SREBP-1c, increased in mice fed WD-CDCA (P<0.001).

Changes in expression of markers of inflammation were scarce (**Table 3**). Expression of tumor necrosis factor α (*Tnfα*) increased more than 2-fold with WD-CA diet (P<0.01) but not with WD-CDCA diet, and expression of Interleukin 1 β (*Il-1β*) remained unchanged.

### Effect of WD-CA and WD-CDCA Diets on Cholesterol Absorption and Gene Expression in the Intestine

**Figure 3:**
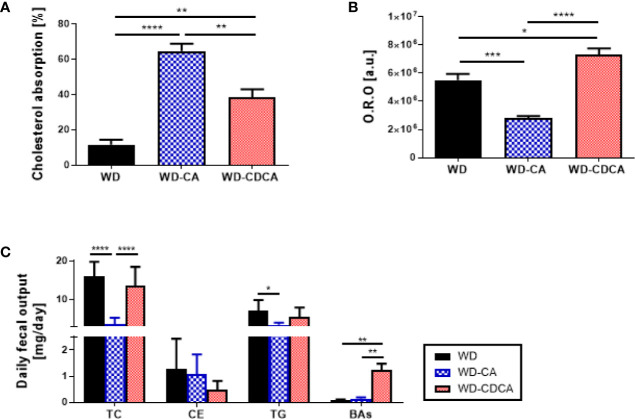
Effect of WD, WD-CA, and WD-CDCA on cholesterol elimination. **(A)** Cholesterol absorption, measured *via* dual isotope incorporation method. **(B)** Quantification of neutral lipid in ileum in arbitrary units [a.u.]. **(C)** Fecal lipid content expressed as daily excretion of TC, cholesterol ester (CE), TG and BAs. Results are presented as mean ± SD, n=6. For statistical difference, *P<0.05, **P<0.01,***P<0.001, ****P<0.0001.

The effect of exogenous CA and CDCA diets on cholesterol absorption was measured using the dual isotope incorporation method (**Figure 3A**). Cholesterol absorption increased more than 5-fold in mice fed WD-CA and 3-fold in mice fed WD-CDCA (P<0.0001). In mice fed with WD-CA, the more than 4-fold reduced daily fecal total cholesterol fecal output (P<0.0001) is due to an increased in cholesterol absorption (P<0.0001) (**Figure 3B**). WD-CA diet also reduced TG elimination with feces by a 2-fold (P<0.05), whereas fecal levels of CE and BAs remained unchanged (**Figure 3B**). In contrast, in mice fed with WD-CDCA, BAs excretion was more than 10-fold higher compared to mice fed with WD (**Figure 3B**).

Cholesterol esters and TG accumulation were assessed in enterocytes by Oil red O staining in the ileum (**Figure 2B**). The intensity of the staining was reduced by 50% with CA and increased by 33% with CDCA (P<0.0001) (**Figure 3C**).

The analysis of gene expression in the intestine is reported in **Table 4**. Interestingly, in the ileum, the genes involved in cholesterol homeostasis were similarly downregulated in mice fed with WD-CA and WD-CDCA, except for *Cyp3a11* which was markedly suppressed only with WD-CDCA. The most dramatic increase was for the molecular targets of *Fxr*, fibroblast growth factor (Fgf15) and SHP*. Fgf15* expression increased 500-fold (P<0.0001) and expression of *Nr0b2* (SHP) increased 1000-fold (P<0.001) (**Table 4**) in mice fed with WD-CA. WD-CDCA diet also increased the expression of these two genes, but to a lesser extent. *Slc51a* (OSTα) expression was upregulated (P<0.01), but only in mice fed WD-CA and the same tendency was observed for *Slc51b* (OSTβ). Expression of all the genes involved in cholesterol efflux, *Abca1*, *Abcg1*, *Abcg5*, *Abcg8*, and *Scarb1* were increased in mice fed with WD-CA, with the biggest increase in *Abca1* expression (P<0.01). This was not observed for animals fed with WD-CDCA, as the only change observed when compared to WD was the reduction of expression of *Abcg1* (P<0.001).

**Table 4:** Effect of CA and CDCA on expression of genes involved in lipid metabolism and inflammation in the intestine.

Role	Gene	WD	WD-CA	WD-CDCA	One way Anova	
		n=6	n=6	n= 6	P
**Cholesterol homeostasis**	*Cyp3a11*	1.08 ± 0.46	1.00 ± 0.70	0.34 ± 0.15*	0.0091
	*Hmgcr*	1.01 ± 0.14	0.63 ± 0.07***	0.79 ± 0.14*	0.0003
	*Srebf2* (SREBP2)	1.01 ± 0.15	0.97 ± 0.19	0.82 ± 0.06	NS
	*Ldlr*	1.03 ± 0.29	0.74 ± 0.14*	0.67 ± 0.13*	0.0158
**BAs signaling**	*Slc51a* (OSTa)	1.01 ± 0.13	2.47 ± 0.71**	1.36 ± 0.52	0.0019
	*Slc51b* (OSTb)	1.09 ± 0.55	1.70 ± 0.47	1.19 ± 0.31	NS
	*Slc10a2* (ASBT)	1.01 ± 0.13	0.78 ± 0.19	0.90 ± 0.23	NS
	*Gpbar1* (TGR5)	1.07 ± 0.51	1.83 ± 0.45	1.12 ± 0.46	NS
	*Fgf15*	1.14 ± 0.57	487 ± 184***	63.9 ± 30.2	0.0001
**Cholesterol transport**	*Abca1*	1.05 ± 0.37	5.18 ± 1.2**	1.16 ± 0.28	0.0018
	*Nr1h3* (LXRα)	1.03 ± 0.17	1.35 ± 0.34	1.17 ± 0.27	NS
	*Abcg1*	1.06 ± 0.45	3.50 ± 0.83***	0.58 ± 0.18**	0.0001
	*Abcg5*	1.01 ± 0.19	2.51 ± 0.67***	1.50 ± 0.19	0.0001
	*Abcg8*	1.02 ± 0.23	3.10 ± 0.65***	1.52 ± 0.29	0.0001
	*Scarb1* (SR-B1)	0.89 ± 0.11	1.74 ± 0.39***	0.64 ± 0.26	0.0001
	*Cav-1*	0.84 ± 0.09	1.66 ± 0.62	0.69 ± 0.39	0.019
	*Npc1l1*	1.02 ± 0.13	0.54 ± 0.05*	1.05 ± 0.07	0.0014
**Nuclear receptors**	*Nr1i2* (PXR)	1.00 ± 0.11	1.75 ± 0.10**	1.29 ± 0.16**	0.0001
	*Nr1h4* (FXR)	1.02 ± 0.19	1.25 ± 0.20***	1.05 ± 0.33	NS
	*Nr0b2* (SHP)	1.08 ± 0.54	1098 ± 589***	11.7 ± 4.6	0.0001
	*Vdr*	1.00 ± 0.11	1.44 ± 0.21**	1.02 ± 0.20	0.003
	*Rxrα*	1.00 ± 0.09	1.41 ± 0.20**	0.99 ± 0.13	0.0007

Results are presented as means ± SD.

For statistical significance, *P < 0.05, **P < 0.01, ***P < 0.001 vs. WD.

NS not significant.

Finally, we analyzed gene expression of the intestinal cholesterol transporter, the transmembrane protein Niemann-Pick C1-Like 1 protein (NPC1L1) in the jejunum. In WD-CA fed mice, there was a significant decrease in mRNA expression when compared to animals fed with WD or WD-CDCA (P<0.05).

### Effect of WD-CA and WD-CDCA Diets on ApoE KO Mice

**Figure 4:**
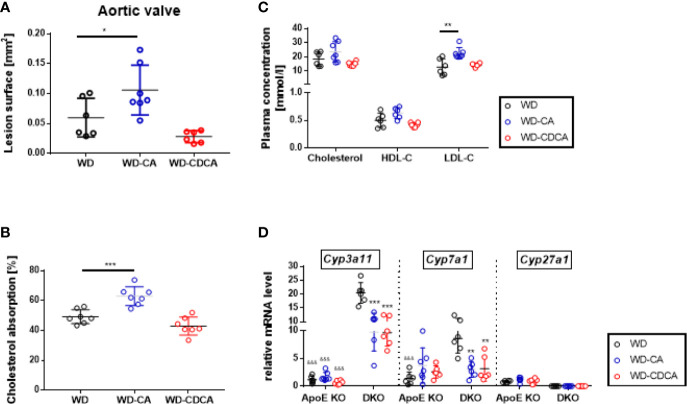
Effect of WD, WD-CA, and WD-CDCA on cardiovascular and metabolic changes in *ApoE* KO mice. Atherosclerotic lesions were quantified in aortic valve sections **(A)**. Cholesterol absorption was measured with dual isotope incorporation **(B)**. Plasma lipid composition **(C)** was measured by chemoluminescence. Cyp3a11, Cyp7a1 and Cyp27a1 mRNA levels were measured in liver **(D)**. Results are presented as means± SD. For statistical difference ***P < 0.001, **P < 0.01, and *P < 0.05; ^&&&^P < 0.001 when compared to DKO mice with the same food regimen.

To investigate the effect of WD-CA and WD-CDCA in mice with normal *Cyp27a1* expression, atherosclerotic lesions were quantified in *ApoE* KO mice fed with WD-CA or WD-CDCA. Similar to DKO mice, WD-CA was pro-atherogenic leading to a 75% increase in atherosclerotic lesions (P<0.05), whereas WD-CDCA had no effect on the size of the atherosclerotic lesions (**Figure 4A**). This phenotype could be explained by a 25% increase in cholesterol absorption specific for WD-CA (P<0.001) (**Figure 4B**) and a different plasma lipid composition (**Figure 4C**), mainly because LDL-C increased by a factor 2 (P<0.01) (**Figure 4C**). Notably, cholesterol absorption in *ApoE* KO mice fed WD alone was almost 5-fold higher than in DKO mice (compare Figs. 4B and 3A). WD-CA had no significant effect on hepatic *Cyp7a1*, *Cyp3a11*, and *Cyp27a1* mRNA levels (**Figure 4D**). Nevertheless, when compared to DKO mice fed with WD-CA and WD-CDCA, the expression of *Cyp3a11* in the liver was still lower in *ApoE* KO (**Figure 4D**). This was not the case for *Cyp7a1* (**Figure 4D**).

## Discussion

In our previous study, *Cyp27a1* deficient mice on *ApoE*^−^*^/^*^−^ background, (DKO mice), fed with WD developed 10-fold less atherosclerosis than their *ApoE* KO littermates (9). Increased cholesterol metabolism *via* over-expression of hepatic *Cyp7a1* and *Cyp3a11* gene expression and reduced cholesterol absorption were proposed as atheroprotective mechanisms. Since feeding *Cyp27a1* KO mice with CA or CDCA reversed the metabolic features of *Cyp27a1* deficient mice (14), we applied a similar treatment to DKO mice fed with WD and re-assessed their phenotype.

**Figure 5:**
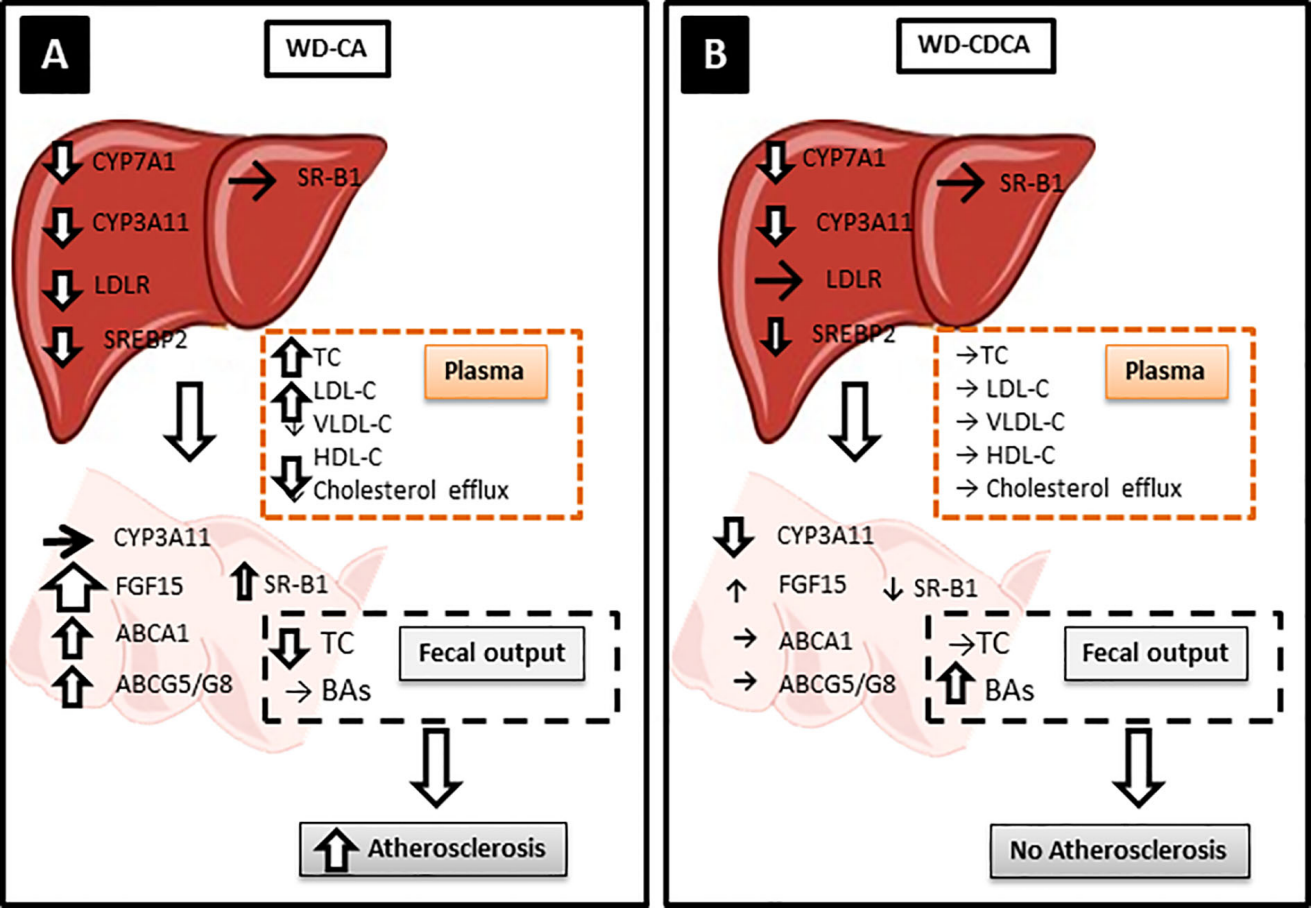
Possible atherosclerotic mechanisms. Hepatic CYP7A1 and CYP3A11 were downregulated to the same level with both diets but only WD-CA was atherogenic. **(A)** In WD-CA fed mice, atherosclerosis was mainly driven by high circulating TC concentrations, low HDL-C and high LDL-C, and reduced cholesterol output in feces. The increased plasma LDL-C was attributed to the reduced LDLR leading to a diminished LDL-C uptake by the liver, driven by a decreased SREPB2. In the intestine, WD-CA promoted the release of FGF15 as well as increased expression of ABCA1, ABCG5, ABCG8, and SR-B1. **(B)** WD-CDCA fed mice failed to develop atherosclerosis, because of low plasma cholesterol, high HDL-C and low LDL-C, the tremendous increase of BAs excretion in feces. In the intestine, WD-CDCA also strongly down regulated CYP3A11, whereas FGF-15 was slightly increased and ABCA1 and ABCG5/G8 were unchanged.

The key findings of our study are summarized in **Figure 5**. Like in *Cyp27a1* KO mice, CA and CDCA down regulated expression of hepatic *Cyp7a1* and *Cyp3a11* to the same extend. However, treatment with exogenous BAs differently modified gene expression in the liver and intestine, plasma lipids composition and cholesterol absorption. This lead to a different hepatic and atherosclerotic phenotype in *Cyp27a1* deficient mice on *ApoE* background fed with WD.

WD-CA fed DKO mice had increased atherosclerotic plaque formation, a finding attributed predominantly to the elevated plasma TC and LDL-C (**Table 1**) combined with increase of intestinal cholesterol absorption and decreased fecal output of cholesterol. The increase in plasma lipids is likely explained by the reduced hepatic expression of *Srebf2* and *Ldlr* (**Table 3**), leading to a decreased LDL uptake by the liver. The measurement of *in vivo* cholesterol absorption and fecal output (**Figures 3A, C**) confirmed an increased cholesterol absorption and reduced fecal output, two elements likely accounting for the pro-atherogenic plasma lipoprotein composition and the atherosclerotic plaque formation. However, we observed increased expression of *Abcg5/g8* and reduced expression of *Npc1l1* in the intestine, a phenotype usually associated with reduced cholesterol absorption (19). This is counterintuitive, but may point to a compensatory response to the changes caused by a different mechanism. Changes in ABCG5/G8 expression were shown to have little impact on plasma cholesterol level (19). Moreover, fecal excretion might have been underestimated, as we did not measure coprostanol and coprostanone. The hepatotoxic effect of CA feeding could also contribute to the diminished hepatic uptake of cholesterol and the development of atherosclerosis. Finally, WD-CA affected the entero-hepatic circulation by increasing massively the expression of *Ffg15* in the intestine.

In contrast to DKO mice fed with WD-CA, DKO mice fed WD-CDCA did not develop atherosclerotic lesions, a phenotype likely attributed to low plasma TC an LDL-C (**Table 1**) combined with decreased intestinal cholesterol absorption and increased BAs excretion (mainly αMCA, βMCA and ωMCA) into feces (**Table 2**). The more hydrophilic composition of bile acid pool as a consequence of accumulation of muricholic acids is highly likely responsible for the lesser induction of cholesterol absorption by WD-CDCA than with WD-CA. With normal levels of hepatic *Ldlr* mRNA (**Table 3**), cholesterol uptake by the liver was not enhanced. Intestinal cholesterol absorption was also preserved, since expression of both *Abca1* and *Abcg5/g8* were not up-regulated (**Table 4**).

The difference in atherosclerotic plaque formation between the two groups of DKO mice could partly be explained by a decrease in reverse cholesterol transport (RCT). In the present study, we analyzed two steps of RCT, first step, the transport of cholesterol to an acceptor in plasma, cholesterol efflux, and the last step, elimination of cholesterol *via* BAs. DKO mice plasma’s acceptor capacity from mice fed with WD-CA was reduced when compared to WD or WD-CDCA, and the efflux correlated to HDL-C. BAs excretion in feces was 10-fold higher with WD-CDCA feeding than with WD-CA feeding. Thus, reduced RCT upon feeding WD-CA may also contribute the formation of atherosclerotic lesions in DKO mice.

The difference in cholesterol absorption between the WD-CA and WD-CDCA fed mice could also be due to the different critical micelle concentration (CMC) of these two BAs. This important physico-chemical parameter reflects the propensity to form miscellar aggregates and to facilitate the transport of liposoluble molecules, such as cholesterol; it is a relevant parameter for evaluation of the biological activity of a BA. With a low CMC, CA is much more efficient than CDCA in facilitating the absorption of dietary cholesterol from WD (26, 27).

FXR is known to play a key role in the regulation of bile acid synthesis and homeostasis. Whereas CA and CDCA are known FXR agonists, MCAs are FXR antagonists (11). Previously, Qi et al. (20) suggested that reducing 12α-hydroxylated BAs and increasing intestinal Tauro-β-MCA, an antagonist of FXR, may reduce high fat diet-induced increase of phospholipids, sphingomyelins and ceramides and ameliorate diabetes and obesity. In this study, we found a very pronounced increase of all 3 MCAs, αMCA, βMCA and ωMCA in feces of mice fed WD-CDCA, pointing indirectly to the importance of these BAs in the prevention of atherosclerosis, whereas in WD-CA fed mice, they remained unchanged.

Interestingly, our treatment did not reverse the hepatomegaly as described by Repa et al. for *Cyp27a1* deficient mice (14), despite the fact that similar doses of CA and CDCA were used. Our DKO mice fed with WD suffered from hepatomegaly with macrovesicular steatosis, and the latter was more severe in WD-CA fed mice. The increase in total cholesterol and cholesteryl ester in the liver (**Table 1**) confirmed this histological observation. In contrast, both liver and the liver/body weight ratio were decreased in DKO mice fed with WD-CDCA (**Table 1**). The difference between Repa’s study and ours can be explained by i) the different background, specifically, *ApoE* deficiency, ii) the high fat and high cholesterol content of the WD and iii) the duration of BAs treatment.

Like in DKO mice, WD-CA was pro-atherogenic in ApoE KO mice with normal *Cyp27a1* expression, mostly because of increased plasma LDL-C and cholesterol absorption. Interestingly, the amount of atherosclerotic plaque in the aortic valve was of the same magnitude in *ApoE* KO and DKO mice fed with WD-CA: they had similar levels of plasma TC, cholesterol absorption and *Cyp7a1* hepatic mRNA. DKO mice fed with WD-CA however had still lower LDL-C and increased hepatic *Cyp3a11* mRNA expression when compared to their *ApoE* KO littermates. This indicates that *Cyp3a11* is still a major key player of the athero-protective mechanism in DKO mice fed with WD-CA. In *ApoE* KO mice fed with WD-CDCA, the amount of atherosclerotic plaque was higher than in DKO mice fed with the same food regimen. This is best explained by the pro-atherogenic lipid profile (higher TC, lower HDL-C and higher LDL-C) and the increased cholesterol absorption in *ApoE* KO mice.

Humans with a loss of function mutation in the CYP27A1 gene suffer from Cerebrotendinous xanthomatosis (CTX). This autosomal recessive disease is characterized by an accumulation of cholesterol, cholestanol and bile alcohols in peripheral tissues, despite normal and slightly decreased circulating total cholesterol and LDL-C concentrations, indicating a defect in RCT. Interestingly, some but not all develop a severe atherosclerosis (21-24). The usual treatment which consists of CDCA 2–3 × 250 mg daily, has no toxic effect on the liver. The variation of development of atherosclerosis in CTX patients could eventually be explained by the relative expression of CYP3A11 and CYP7A1, but it remains to be proven.

One limitation of the study is that we could not measure bile alcohols, cholestetanol and oxysterols in plasma because of the limitation of size sample. A second limitation is that we based our conclusions on assessment of mRNA levels alone. Posttranscriptional events that may be relevant for the biological actions have not been considered here.

In conclusion, experimental treatment of CYP27A1 deficiency with two different BAs had very different metabolic consequences and cardiovascular outcomes, demonstrating that current view of how BA infusion works is simplistic. A better understanding of exact mechanisms of how BAs regulate lipid metabolism and if BAs have a direct effect on gene expression in macrophages is essential for development of future therapies involving BAs.

## Data Availability Statement

The raw data supporting the conclusions of this article will be made available by the authors, without undue reservation.

## Ethics Statement

The animal study was reviewed and approved by Ethics Committee for Animal Experiments of the Veterinary Administration of the Canton of Berne, Switzerland.

## Author Contributions

LZ conducted experiments and participated in the drafting of the manuscript. DS analyzed the results and participated in the editing of the manuscript. BV helped with the interpretation of the results. GE conceived and designed the experiments and wrote the manuscript. GE is the guarantor of this work. All authors contributed to the article and approved the submitted version.

## Conflict of Interest

The authors declare that the research was conducted in the absence of any commercial or financial relationships that could be construed as a potential conflict of interest.

## References

[1] Norlin M von Bahr S Bjorkhem I Wikvall K On the substrate specificity of human CYP27A1: implications for bile acid and cholestanol formation. J Lipid Res (2003) 44(8):1515–22. 10.1194/jlr.M300047-JLR200 12777473

[2] Cali JJ Hsieh CL Francke U Russell DW Mutations in the bile acid biosynthetic enzyme sterol 27-hydroxylase underlie cerebrotendinous xanthomatosis. J Biol Chem (1991) 266(12):7779–83. PMC4449724 2019602

[3] Bjorkhem I Mechanism of degradation of the steroid side chain in the formation of bile acids. J Lipid Res (1992) 33(4):455–71. 1527470

[4] Russell DW Setchell KD Bile acid biosynthesis. Biochemistry (1992) 31(20):4737–49. 10.1021/bi00135a001 1591235

[5] Qi Y Jiang C Cheng J Krausz KW Li T Ferrell JM Bile acid signaling in lipid metabolism: metabolomic and lipidomic analysis of lipid and bile acid markers linked to anti-obesity and anti-diabetes in mice. Biochim Biophys Acta (2015) 1851(1):19–29. 10.1016/j.bbalip.2014.04.008 24796972 PMC4219936

[6] Escher G Krozowski Z Croft KD Sviridov D Expression of sterol 27-hydroxylase (CYP27A1) enhances cholesterol efflux. J Biol Chem (2003) 278(13):11015–9. 10.1074/jbc.M212780200 12531903

[7] Mukhamedova N Escher G D’Souza W Tchoua U Grant A Krozowski Z Enhancing apolipoprotein A-I-dependent cholesterol efflux elevates cholesterol export from macrophages in vivo. J Lipid Res (2008) 49(11):2312–22. 10.1194/jlr.M800095-JLR200 PMC5475370 18622028

[8] Babiker A Andersson O Lund E Xiu RJ Deeb S Reshef A Elimination of cholesterol in macrophages and endothelial cells by the sterol 27-hydroxylase mechanism. Comparison with high density lipoprotein-mediated reverse cholesterol transport. J Biol Chem (1997) 272(42):26253–61. 10.1074/jbc.272.42.26253 9334194

[9] Hall E Hylemon P Vlahcevic Z Mallonee D Valerie K Avadhani N Overexpression of CYP27 in hepatic and extrahepatic cells: role in the regulation of cholesterol homeostasis. Am J Physiol Gastrointest Liver Physiol (2001) 281(1):G293–301. 10.1152/ajpgi.2001.281.1.G293 11408283

[10] Zurkinden L Solca C Vogeli IA Vogt B Ackermann D Erickson SK Effect of Cyp27A1 gene dosage on atherosclerosis development in ApoE-knockout mice. FASEB J Off Publ Fed Am Soc Exp Biol (2014) 28(3):1198–209. 10.1096/fj.13-233791 PMC4046167 24327605

[11] Russell DW The enzymes, regulation, and genetics of bile acid synthesis. Annu Rev Biochem (2003) 72:137–74. 10.1146/annurev.biochem.72.121801.161712 12543708

[12] Ikeda I Factors affecting intestinal absorption of cholesterol and plant sterols and stanols. J Oleo Sci (2015) 64(1):9–18. 10.5650/jos.ess14221 25742922

[13] Duan Y Zhang F Yuan W Wei Y Wei M Zhou Y Hepatic cholesterol accumulation ascribed to the activation of ileum Fxr-Fgf15 pathway inhibiting hepatic Cyp7a1 in high-fat diet-induced obesity rats. Life Sci (2019) 232:116638. 10.1016/j.lfs.2019.116638 31288013

[14] Chiang JYL Ferrell JM Bile Acids as Metabolic Regulators and Nutrient Sensors. Annu Rev Nutr (2019) 39:175–200. 10.1146/annurev-nutr-082018-124344 31018107 PMC6996089

[15] Wang H Chen J Hollister K Sowers LC Forman BM Endogenous bile acids are ligands for the nuclear receptor FXR/BAR. Mol Cell (1999) 3(5):543–53. 10.1016/S1097-2765(00)80348-2 10360171

[16] Song P Rockwell CE Cui JY Klaassen CD Individual bile acids have differential effects on bile acid signaling in mice. Toxicol Appl Pharmacol (2015) 283(1):57–64. 10.1016/j.taap.2014.12.005 25582706 PMC7748369

[17] Gonzalez FJ Jiang C Xie C Patterson AD Intestinal Farnesoid X Receptor Signaling Modulates Metabolic Disease. Dig Dis (2017) 35(3):178–84. 10.1159/000450908 PMC6595218 28249275

[18] Li-Hawkins J Gafvels M Olin M Lund EG Andersson U Schuster G Cholic acid mediates negative feedback regulation of bile acid synthesis in mice. J Clin Invest (2002) 110(8):1191–200. 10.1172/JCI0216309 PMC150802 12393855

[19] Ourlin JC Lasserre F Pineau T Fabre JM Sa-Cunha A Maurel P The small heterodimer partner interacts with the pregnane X receptor and represses its transcriptional activity. Mol Endocrinol (2003) 17(9):1693–703. 10.1210/me.2002-0383 12805410

[20] Inagaki T Choi M Moschetta A Peng L Cummins CL McDonald G Fibroblast growth factor 15 functions as an enterohepatic signal to regulate bile acid homeostasis. Cell Metab (2005) 2(4):217–25. 10.1016/j.cmet.2005.09.001 16213224

[21] Repa JJ Lund EG Horton JD Leitersdorf E Russell DW Dietschy JM Disruption of the sterol 27-hydroxylase gene in mice results in hepatomegaly and hypertriglyceridemia. Reversal by cholic acid feeding. J Biol Chem (2000) 275(50):39685–92. 10.1074/jbc.M007653200 11001949

[22] Keren Z Falik-Zaccai TC Cerebrotendinous xanthomatosis (CTX): a treatable lipid storage disease. Pediatr Endocrinol Rev (2009) 7(1):6–11. 19696711

[23] Quattropani C Vogt B Odermatt A Dick B Frey BM Frey FJ Reduced activity of 11 beta-hydroxysteroid dehydrogenase in patients with cholestasis. J Clin Invest (2001) 108(9):1299–305. 10.1172/JCI12745 PMC209437 11696574

[24] Locket PL Gallaher DD An improved procedure for bile acid extraction and purification and tissue distribution in the rat. Lipids (1989) 24(3):221–3. 10.1007/BF02535238 2761355

[25] Pecks U Mohaupt MG Hutten MC Maass N Rath W Escher G Cholesterol acceptor capacity is preserved by different mechanisms in preterm and term fetuses. Biochim Biophys Acta (2013) 1841(2):251–8. 10.1016/j.bbalip.2013.11.008 24291326

[26] Reynier MO Montet JC Gerolami A Marteau C Crotte C Montet AM Comparative effects of cholic, chenodeoxycholic, and ursodeoxycholic acids on micellar solubilization and intestinal absorption of cholesterol. J Lipid Res (1981) 22(3):467–73. 7240971

[27] Wang DQ Cohen DE Carey MC Biliary lipids and cholesterol gallstone disease. J Lipid Res (2009) 50 Suppl:S406–11. 10.1194/jlr.R800075-JLR200 PMC2674701 19017613

