# Supplementary material for: Downregulation of Cyp7a1 by Cholic Acid and Chenodeoxycholic Acid in Cyp27a1/ApoE Double Knockout Mice: Differential Cardiovascular Outcome
Source: Front Endocrinol (Lausanne). 2020 Oct 28;11:586980. doi: 10.3389/fendo.2020.586980 (PMC7656987; doi:10.3389/fendo.2020.586980)
Supplement: Supplementary file 1 [file Table_1.pdf]

SUPPLEMENTARY TABLE 1. Primers and probes for qPCR, using the Universal ProbeLibrary (Roche)

Gene ID	Accession number	Primers	Probe
Abcc2	NM_013806.2	5'-caaatccaattctctacctatgcac-3'<br>5'-gcctgcagtgttggatca-3'	92
Abcg1	NM_009593.2	5'-gggtctgaactgccctacct-3'<br>5'-tactcccctgatgccacttc-3'	3
Slc10a2	NM_011388.2	5'-gactagctggtcaaccctggta-3'<br>5'-gggggagaaggagagctgta-3'	22
Cav-1	NM_007616.4	5'-ccagggaaacctcctcaga-3'<br>5'-ccggatgggaacagtgtaga-3'	71
Npc1l1	NM_207242.2	5'-caacatcttcatctttgttcttgag-3'<br>5'-gccaatgtgagcctctcg-3'	110
Slc10a1	NM_011387.2	5'-atgaagggggacatgaacct-3'<br>5'-gtagatgtataagaggagaggcatca-3'	78
Slco1a1	NM_020495.1	5'-cccgtgactaatccaacaaca-3'<br>5'-gcttctcagagaccatagaaaacc-3'	51
Slc61a	NM_145932.3	5'-gctgcccacctctcatactt-3'<br>5'-gaagaaggcgtactggaaagg-3'	18
Slc51b	NM_178933.2	5'-gagcatcctggcaaacaga-3'<br>5'-tgcaggtcttctggtgtttct-3'	5
Vdr	NM_009504.4	5'-cacctggctgatcttgtcagt-3'<br>5'-ctggtcatcagaggtgaggtc-3	89
Rxrα	NM_011305.3	5'-acatgcagatggacaagacg-3'<br>5'-gggtttgagagccccttaga-3'	26

Pparϒ	NM_008904.2	5'-tgaaagggccaaacagagag-3'<br>5'-gtaaatcacacggcgctctt-3'	29
Tnfα	NM_013693.3	5'-ctgtagcccacgtcgtagc-3'<br>5'-ttgagatccatgccgttg-3'	25
Il6	NM_031168.2	5'-gctaccaaactggatataatcagga-3'<br>5'-ccaggtagctatggtactccagaa-3'	6
